# Social media misinformation during the COVID-19 pandemic: Impacts on public mental health

**DOI:** 10.1192/j.eurpsy.2021.133

**Published:** 2021-08-13

**Authors:** M. Alvarez-Mon

**Affiliations:** Department Of Psychiatry, University Hospital Infanta Leonor, Madrid, Spain

**Keywords:** Twitter, COVID

## Abstract

Social Media Misinformation During the COVID-19 Pandemic: Impacts on Public Mental Health

**Introduction:**

Some behavioral measures such as handwashing, masking or social distancing are among the most effective tools to combat COVID-19 pandemic.

**Objectives:**

Describe the extent to which major media outlets in the United States and Spain have tweeted about COVID-19 health related behaviors, and determine if differences exist between major media outlets in the two countries.

**Methods:**

We analyzed contents posted on Twitter by 25 major media outlet’s (15 from USA and 10 from Spain) about COVID health related behaviors (HRB). News content were analyzed and classified as well as Twitter users’ reactions.

**Results:**

Masking and quarantine were the HRB that generated most of the tweets. However, we found differences between media outlets in the two countries. Twitter user´s engaged more with tweets posted by USA media. Most of the tweets describing HRB from the general population were consistent with CDC/WHO guidelines.

**Conclusions:**

Understanding the public view of these HRB is necessary to design promotional strategies aimed at the appropriate population.

**Disclosure:**

No significant relationships.

